# Supplementary material for: Rheological and Mechanical Properties of Silica/Nitrile Butadiene Rubber Vulcanizates with Eco-Friendly Ionic Liquid
Source: Polymers (Basel). 2020 Nov 23;12(11):2763. doi: 10.3390/polym12112763 (PMC7700482; doi:10.3390/polym12112763)
Supplement: Supplementary file 1 [file polymers-12-02763-s001.pdf]

polymers

Article

Rheological and mechanical properties of silica/nitrile butadiene rubber vulcanizates with eco-friendly ionic liquid

Munir Hussain [1], Sohail Yasin [1], Hafeezullah Memon [2], Zhiyun Li [1], Xinpeng Fan[1], Muhammad Adnan Akram [1], Wanjie Wang [3] Yihu Song [1,*], and Qiang Zheng [1]

[1] MOE Key Laboratory of Macromolecular Synthesis and Functionalization, Department of Polymer Science and Engineering, Zhejiang University, Hangzhou 310027, China; munir88@zju.edu.cn (M.H.); soh.yasin@gmail.com (S.Y.); yunlemon@zju.edu.cn (Z.L); fxpolymer@zju.edu.cn (X.F); m.adnanansari232@zju.edu.cn (M.A.A); zhengqiang@zju.edu.cn (Q.Z)
[2] College of Textile Science and Engineering (International Institute of Silk), Zhejiang Sci-Tech University, Hangzhou,310018, China; hm@zstu.edu.cn (H.M)
[3] College of Materials Science and Engineering, Henan Key Laboratory of Advanced Nylon Materials and application, Zhengzhou University, Zhengzhou 450001, China; wwj@zzu.edu.cn (W.W)
* Correspondence: s_yh0411@zju.edu.cn (Y.S)

Supporting Information

1.Thermogravimetric analysis

Thermal stability of the vulcanizates was investigated with help of thermogravimetric analysis (TGA, Q1000, TA Instruments, USA). Samples of 5-10 mg weight were pre-heated for 15 min at 100 °C and were heated to 900 °C at a rate of 10 °C /min under a nitrogen atmosphere with a flow rate of 50 mL/min.

2. Modulated differential scanning calorimetry

Modulated differential scanning calorimetry was carried out to measure glass transition temperature (T_g) of NBR in the nanocomposites by using a differential scanning calorimeter (Discovery 25, TA Instruments, USA) under a temperature-modulated mode. Before measurement, a calibration for determining the accurate heat capacity of sapphire was performed with the same procedure. During the measurement, the samples of 5-10 mg wrapped in aluminium pans were cooled to -50 °C at 1 °C/min to avoid physical aging. After equilibrating for 5 min, the samples heat capacity was measured at heating rate 1 °C/min where the time and amplitude of modulated signal were set as 120 s and ± 1°C.

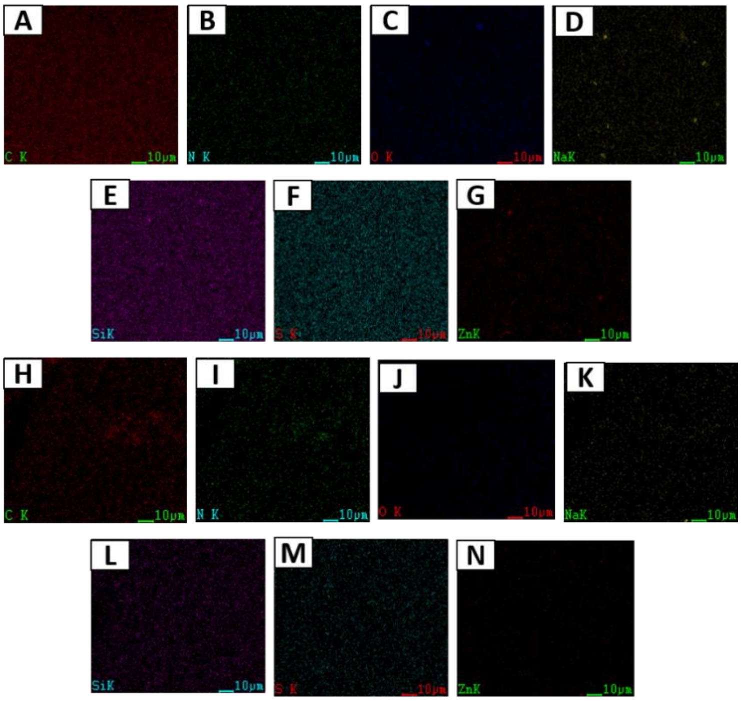

Figure S1. SEM-EDS mapping of elements of carbon (A and H), nitrogen (B and I), oxygen (C and J), sodium (D and K), silicon (E and L), sulphur (F and M) and zinc elements (G and N) for Si69- or Si69/[EMIM]OAc containing vulcanizates (φ = 0.041).

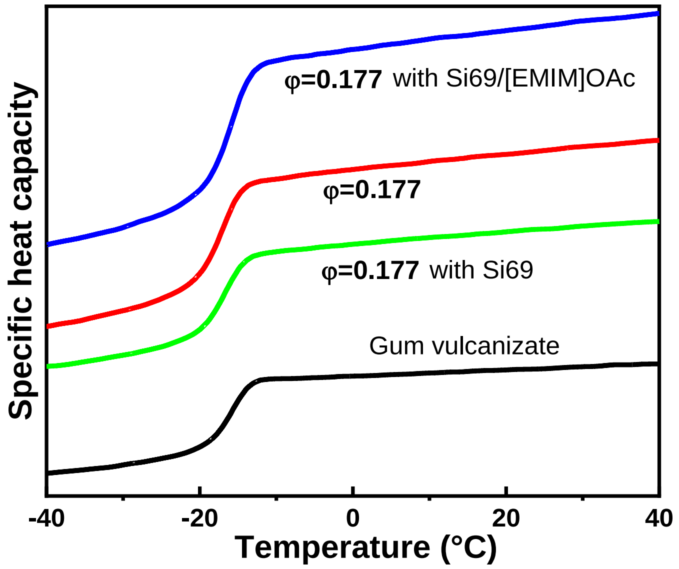

Figure S2. Specific heat capacity of the gum vulcanizate (φ = 0) and its nanocomposites (φ = 0.177). The curves are shifted vertically for clear display.

Table S1: Glass transition temperature (T_g) of the gum vulcanizate (φ = 0) and its nanocomposites (φ = 0.177).

**Coupling agent**	φ	T_g **(°C)**
/	0	-16.2
/	0.177	-16.4
Si69	0.177	-15.4
Si69/[EMIM]OAc	0.177	-14.7

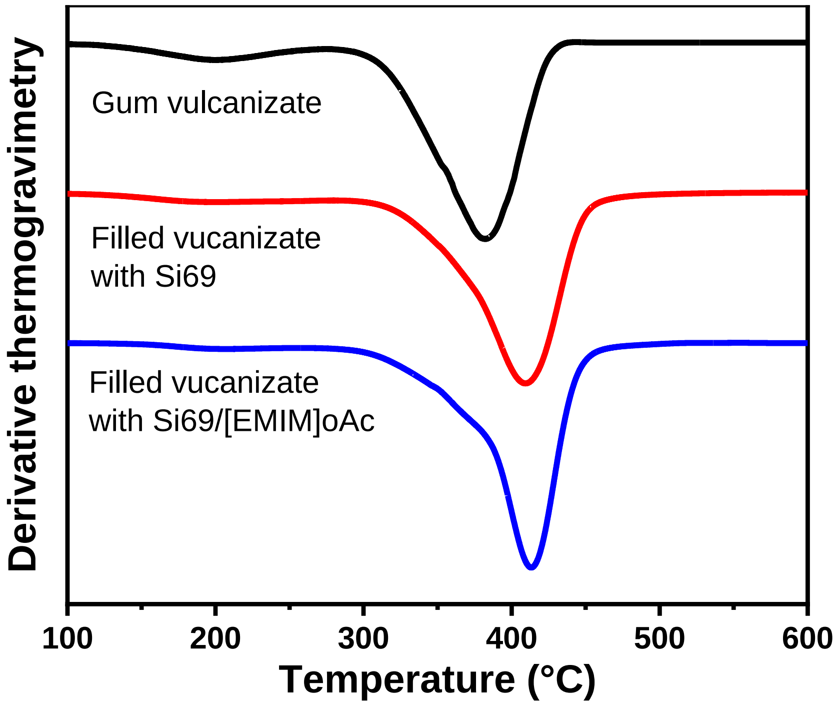

Figure S3. Derivative thermogravimetry curves of the gum vulcanizate (φ = 0) and its Si69- or Si69/[EMIM]OAc-containing nanocomposites (φ = 0.177) in nitrogen atmosphere. The curves are shifted vertically for clear display.

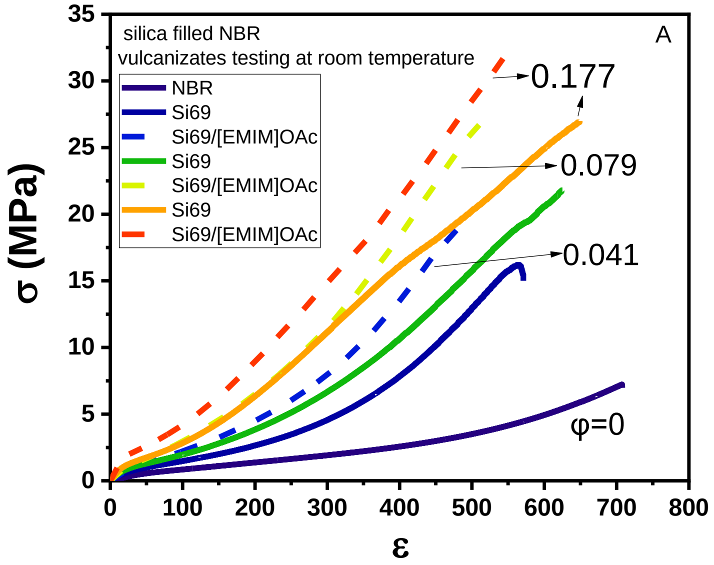

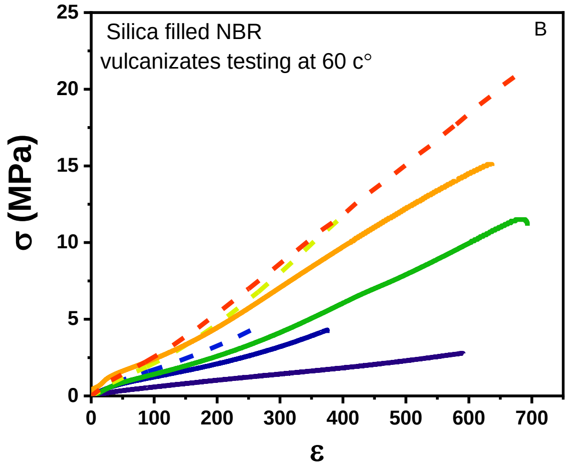

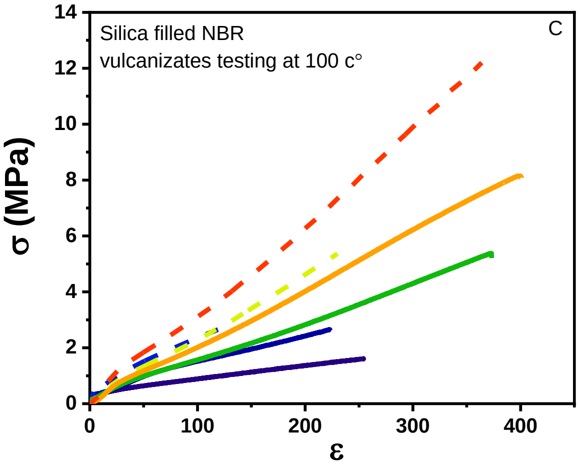

Figure S4. Stress-strain curves for Si69- or Si69/[EMIM]OAc-containing nanocomposites at room temperature (A), 60 °C(B) and 100 °C (C).
